# The synthesis, crystal structure and Hirshfeld analysis of 4-(3,4-di­methyl­anilino)-*N*-(3,4-di­methyl­phen­yl)quinoline-3-carboxamide

**DOI:** 10.1107/S2056989020000298

**Published:** 2020-01-17

**Authors:** Ligia R. Gomes, John Nicolson Low, Fernanda Borges, Alexandra Gaspar, Francesco Mesiti

**Affiliations:** a REQUIMTE, Departamento de Química e Bioquímica, Faculdade de Ciências da Universidade do Porto, Rua do Campo Alegre, 687, P-4169-007, Porto, Portugal; b FP-ENAS-Faculdade de Ciências de Saúde, Escola Superior de Saúde da UFP, Universidade Fernando Pessoa, Rua Carlos da Maia, 296, P-4200-150 Porto, Portugal; c Department of Chemistry, University of Aberdeen, Meston Walk, Old Aberdeen, AB24 3UE, Scotland; d CIQUP Departamento de Quιmica e Bioquιmica, Faculdade de Ciências, Universidade do Porto, 4169-007 Porto, Portugal; e Department of "Scienze della Vita", University "Magna Graecia" of Catanzaro, Catanzaro, Italy

**Keywords:** crystal structure, hydrogen bonding, quinoline, carboxamide, amine

## Abstract

The structure of the title quinoline carboxamide derivative, C_26_H_25_N_3_O, is described. The quinoline moiety is not planar as a result of a slight puckering of the pyridine ring. The secondary amine has a slightly pyramidal geometry, certainly not planar. Both intra- and inter­molecular hydrogen bonds are present. Hirshfeld surface analysis and lattice energies were used to investigate the inter­molecular inter­actions.

## Abstract 2

The quinoline moiety of the title quinoline carboxamide derivative is not planar as a result of a slight puckering of the pyridine ring. The secondary amine has a slightly pyramidal geometry.

## Chemical context   

Quinoline (1-aza-naphthalene or benzo[b]pyridine) is a natural heterocyclic building block often used as a template for derivatization and generation of drug-like libraries for the discovery of novel bioactive ligands (Mugnaini *et al.*, 2009 ▸; Musiol, 2017 ▸). Quinoline-based compounds are well known for their anti­malarial activity (Antony & Parija, 2016 ▸), although a large spectrum of other biological activities, such as anti­cancer, anti­microbial, anti-inflammatory, anti­oxidant, anti­hypertensive and against neurodegenerative diseases, have also been ascribed to these types of heterocyclic compounds (Nainwal *et al.*, 2019 ▸).

This work is a continuation of our investigation into the preparation, structural analysis and pharmacological properties of substituted heterocyclics including, for example, new insights in the discovery of novel h-MAO-B inhibitors obtained by the structural characterization of a series of *N*-phenyl-4-oxo-4*H*-chromene-3-carboxamide derivatives (Gomes *et al.*, 2015*a* ▸). Other chromone and coumarin carboxamides are discussed in Gomes *et al.* (2015*b* ▸, 2016 ▸).
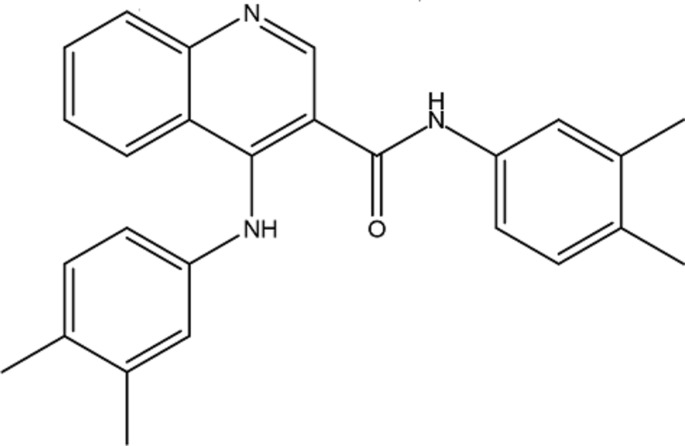


Here we report the synthesis and structural characterization of a quinoline-3-carboxamide derivative, 4-(3,4-di­methyl­anilino)-*N*-(3,4-di­methyl­phen­yl)quinoline-3-carboxamide, **1**.

## Structural commentary   

**Figure 1:**
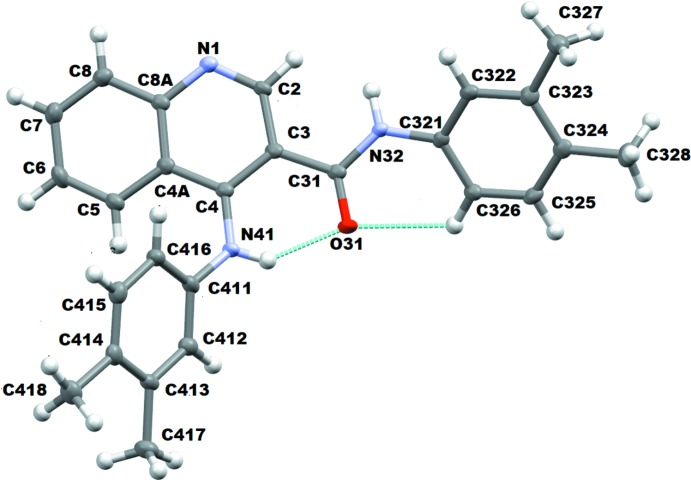
A view of the asymmetric unit of **1** with the atom-numbering scheme. Displacement ellipsoids are drawn at the 50% probability level.

An ellipsoid plot for compound **1** is shown in Fig. 1 ▸. The quinoline ring system is not planar, with atoms C2 and C4 deviating from the mean plane of the quinoline ring by −0.110 (3) and 0.125 (3) Å, respectively, and C6 lying −0.100 (3) Å below the mean plane. The pyridine ring is slightly puckered with a screw-boat conformation, *Q* = 0.087 (3)Å, θ = 106 (2)° and φ = 25 (2)°. The mean plane of this ring makes a dihedral angle of 7.49 (13)° with the mean plane of the benzene ring of the quinoline moiety. The angles between the mean planes of the quinoline ring and the benzene rings with pivot atoms C321 and C411 are 28.99 (11) and 59.16 (11)° respectively. The dihedral angle between the mean plane of these benzene rings is 64.71 (14)°.

The amide group attached to C3 is coplanar with the quinoline ring system. The C—N rotamer of the amide has an *anti* conformation placing the quinoline ring *trans* in relation to the ring with pivot atom C321. The amide group atoms are essentially coplanar with the quinoline ring with deviations of −0.034 (3), (C31), −0.009 (2) (O31), 0.009 (2), (N32) and 0.145 (3) Å (C321). The geometric arrangement of the amide permits the formation of an intra­molecular hydrogen bond between the amine hydrogen atom and the carboxyl group of the amide, N41—H41⋯O31; geometric parameters are given in Table 1 ▸. A further intra­molecular hydrogen bond, C326—H326⋯O31, occurs.

The secondary amine has a slightly pyramidal geometry, certainly not planar. The angles C411—N41—C4, C41—N41—H41 and C411—N41—H41 are 125.7 (2), 112 (2) and 115 (2)°, respectively, the sum of which (352.7°) is less than 360°; in addition, atom H41 lies 0.41 (3) Å out of the C4/N41/C411 mean plane, confirming the *sp* ^3^ hybridization of N41. An inspection of the amine bond lengths shows that there is a slight asymmetry of the electronic distribution around it: C4—N41 = 1.364 (3) Å while N41—C411 = 1.437 (4) Å, suggesting there is higher density between the nitro­gen and the carbon atom of the quinoline ring system. However, these bonds and angles are typical for a C_quinoline_–NH–C–*R* group, see the *Database Survey* below. As a consequence of the screw-boat pucker of the pyridine ring, the C4—N41 bond is displaced from the pyridine mean plane with a deviation of 0.159 (2) Å for N41; atom C411 is displaced by 0.965 (3) Å and consequently, the N41—C411 bond lies further from the mean plane.

## Supra­molecular features   

**Figure 2:**
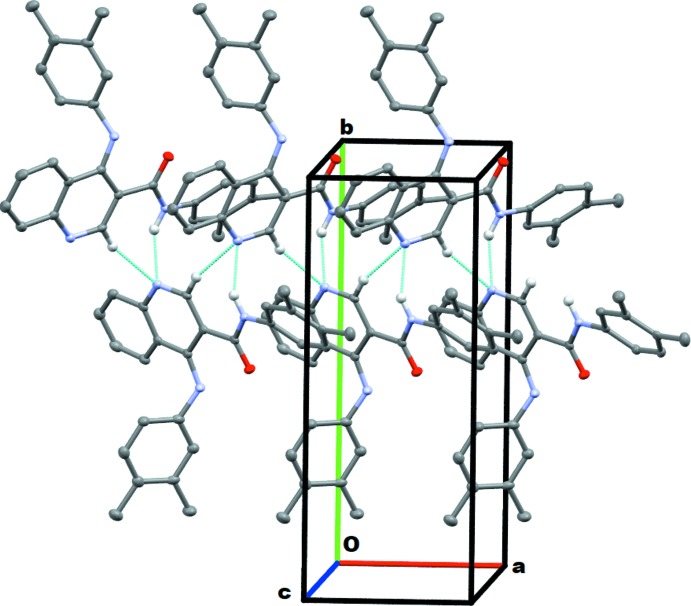
A view of the N32—H32⋯N1 *C*6 chain running along the *a* axis with the supplementary C2—H2⋯N1 bond. Hydrogen atoms not involved in the hydrogen bonding are omitted for clarity.

In the crystal, the mol­ecules are linked by N32—H32⋯N1(*x* + 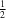, −*y* + 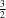, −*z* + 1), hydrogen bonds, forming *C*6 chains which run parallel to the *a*-axis formed by the action of the 2_1_ screw axis at (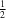, 0, 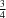). This is supplemented by the weak C2—H2⋯N1(*x* + 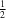, −*y* + 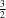, −*z* + 1) hydrogen bond, Table 1 ▸ and Fig. 2 ▸. The other weak hydrogen bonds, C416—H416⋯O31 and C418—H41*B*⋯O31, both involve atom O31 as an acceptor and link the chains described above to form a sheet which extends along the *b*-axis direction.

No π–π inter­actions occur but there is a possible C—H⋯π inter­action, C326—H326⋯*Cg*, involving the pyridine ring (Table 1 ▸), which is discussed more fully below.

## Hirshfeld surface analysis and lattice energies   

**Figure 3:**
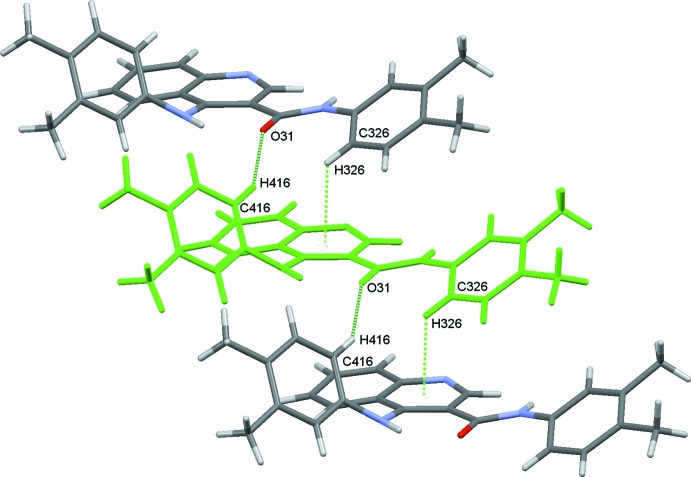
Mol­ecule pairs **Ia**/**Ib**: *x* − 1, *y*, *z* (top) and *x* + 1, *y*, *z* (bottom). Values of energies by pair: *E*_tot_ = −55.9 kJ mol^−1^, *E*_coul_ = −21.4 kJ mol^−1^, *E*_pol_ = −10.0 kJ mol^−1^, *E*_disp_ = −79.5 kJ mol^−1^ and *E*_rep_ = 55.0 kJ mol^−1^. Inter­action energies were calculated using *PIXEL3.1* (Gavezzotti, 2003 ▸, 2008 ▸) based on densities computed with G09 using the mp2/6–31** level of theory.

Hirshfeld surfaces (McKinnon *et al.*, 2004 ▸) and two-dimensional fingerprint (FP) plots provide complementary information concerning the inter­molecular inter­actions discussed above. The analyses were generated using *Crystal Explorer 3.1* (Wolff *et al.*, 2012 ▸). The lattice energies for **1** were analysed after performing calculations as implemented in the *PIXEL* program (Gavezzotti, 2003 ▸, 2008 ▸). The total stabilization energy of the crystal packing, *E*_tot_ is −207.0 kJ mol^−1^, distributed as Coulombic, (*E*_coul_ = −112.9 kJ mol^−1^), polarization (*E*_pol_ = −52.8 kJ mol^−1^), dispersion (*E*_disp =_ −251.6 kJ mol^−1^) and repulsion (*E*_rep_ = 210.4 kJ mol^−1^). The dispersive energy contributes the most to the total stabilization energy of the lattice, in addition to the C—H⋯O hydrogen bonds, and to the C—H⋯π inter­action. The stabilization energy comes from six sub-structural motifs made by the mol­ecule pairs **I** to **VI** that are shown in Figs. 3 ▸ to 8, together with the symmetry codes as well as the respective energies. They contribute a total energy of −369.4 kJ mol^−1^ for the lattice, half of it, −184.7 kJ mol^−1^ attributed to the (*x, y, z*) mol­ecule. That energy corresponds approximately to 88% of the total stabilization energy of the network.

The percentages of atom⋯atom close contacts taken from the FP plot (McKinnon *et al.*, 2004 ▸) for **1** shows that, apart from the H⋯H contacts (58.4%), there are high percentages of C⋯H/H⋯C close contacts (27.0%) and of N⋯H/H⋯N close contacts (6.5%), see Table 2 ▸.

Apart from the intra­molecular hydrogen bond with N41, the carboxyl oxygen atom O31 involves its lone pairs in another two inter­molecular C—H⋯O inter­actions, O31⋯H416—C416 and O31⋯H41*B*—C418. The first inter­action creates chains running along the *a*-axis direction that are further stabilized by C—H⋯π inter­actions (C326—H326⋯*Cg*_pyridine_), as can be identified by the red spots in the Hirshfeld Surface (McKinnon *et al.*, 2004 ▸) for the mol­ecule, Fig. 9 ▸, and they form two mol­ecule pairs, identified as sub-structures **Ia/Ib** in Fig. 3 ▸. Each of those pairs contribute −55.9 kJ mol^−1^ to the stabilization of the lattice, mainly dispersion energy. The second inter­action, O31⋯H41*B*—C418, makes another two mol­ecule pairs, **IIIa**/**IIIb**, Fig. 5 ▸. In this substructure the Coulombic energy is higher than the dispersive energy, which is indicative of the minor importance of the inter­actions involving the aromatic rings. These hydrogen bonds can also be identified as red spots in the HS, Fig. 9 ▸.

**Figure 4:**
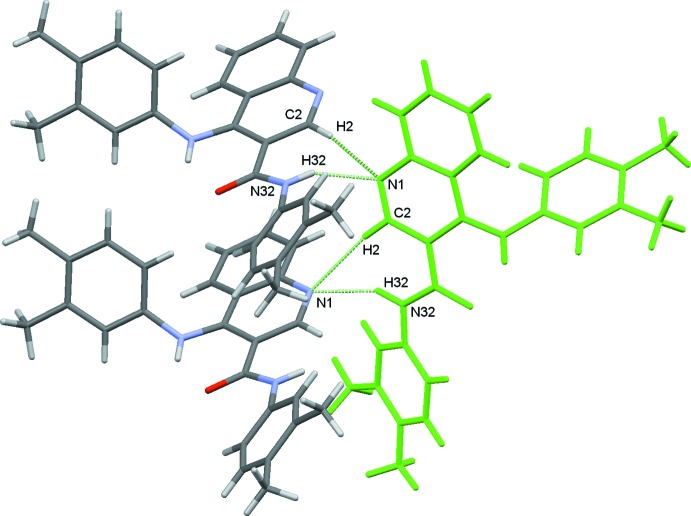
Mol­ecule pairs **IIa**/**IIb**: *x* − 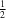, –*y* + 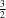, −*z* + 1 (top) and *x* − 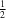, −*y* + 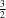, −*z* + 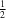, –*y* + 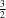, −*z* + 1 (bottom). Values of energies by pair: *E*_tot_ = −52.3 0 kJ mol^−1^, *E*_coul_ = −59.10 kJ mol^−1^, *E*_pol_ = −26.9 0 kJ mol^−1^, *E*_disp_ = −41.5 0 kJ mol^−1^ and *E*_rep_ = 75.2 0 kJ mol^−1^. Inter­action energies were calculated using *PIXEL3.1* (Gavezzotti, 2003 ▸, 2008 ▸) based on densities computed with G09 using the mp2/6–31** level of theory.

**Figure 5:**
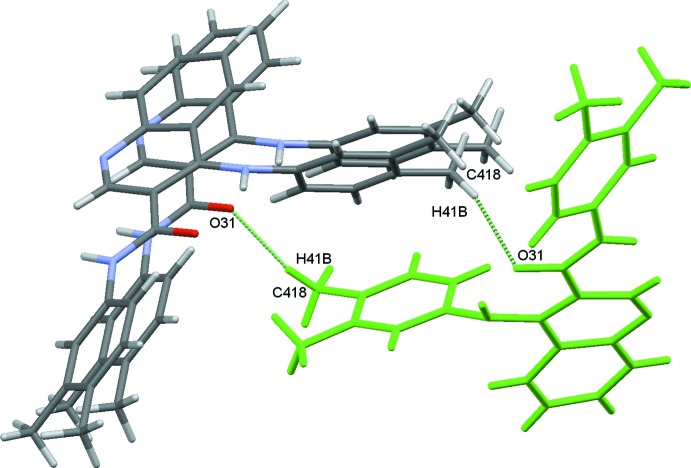
Mol­ecule pairs **IIIa**/**IIIb**: (*x* + 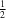, −*y* + 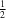, *z +* 1 (top) and *x* − 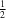, −*y* + 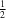, *z* + 1 (bottom). Values of energies by pair: *E*_tot_ = −30.0 kJ mol^−1^, *E*_coul_ = −11.3 kJ mol^−1^, *E*_pol_ = −4.5 kJ mol^−1^, *E*_disp_ = −36.0 kJ mol^−1^, *E*_rep_ = 21.8 kJ mol^−1^. Inter­action energies were calculated using *PIXEL3.1* (Gavezzotti, 2003 ▸, 2008 ▸) based on densities computed with G09 using the mp2/6–31** level of theory.

The nitro­gen atom N32 acts as a donor for N1 (N32—H32⋯N1). N1 also acts as an acceptor for C6, making a C6—H6⋯N1 hydrogen bond, seen as a red spot in Fig. 9 ▸. Those inter­actions give sub structural motifs **IIa/IIb**, Fig. 4 ▸. The mol­ecules are linked by N32—H32⋯N1(*x* + 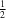, −*y* + 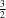, −*z* + 1) hydrogen bonds, forming *C*6 chains which run parallel to the *a*-axis direction, formed by the action of the 2_1_ screw axis at (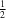, 0, 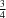). This is supplemented by the weak C2—H2⋯N1(*x* + 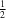, −*y* + 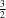, −*z* + 1) hydrogen bond, Figs. 3 ▸ and 4 ▸.

**Figure 6:**
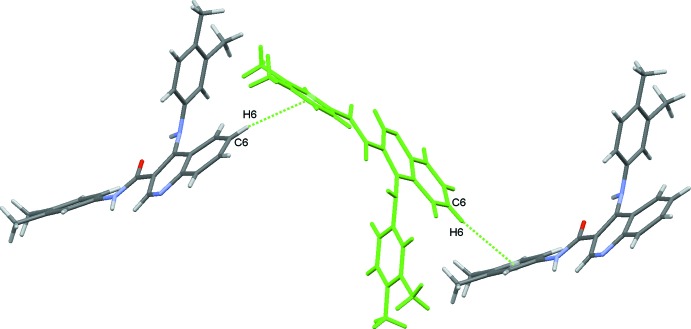
Mol­ecule pairs **IVa/IVb**: −*x* + 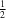, −*y* + 1, *z* + 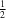 (left) and −*x* + 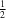, −*y* + 1, *z* − 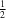 (right). Values of energies by pair: *E*_tot_ = −20.7 kJ mol^−1^, *E*_coul_ = −7.4 kJ mol^−1^, *E*_pol_ = −4.4 kJ mol^−1^, *E*_disp_ = −31.3 kJ mol^−1^, *E*_rep_ = 22.5 kJ mol^−1^. Inter­action energies were calculated using *PIXEL3.1* (Gavezzotti, 2003 ▸, 2008 ▸) based on densities computed with G09 using the mp2/6–31** level of theory.

In addition, the C—H⋯π inter­action can also be identified in the HS of the mol­ecule, Fig. 9 ▸. The inter­action connects the mol­ecules in zigzag chains running along the *c*-axis direction, as a result of the propagation of the mol­ecule pairs **IVa**/**IVb** depicted in Fig. 6 ▸.

**Figure 7:**
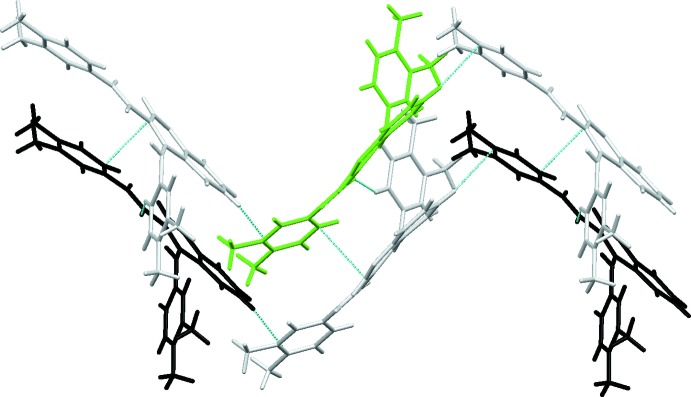
Mol­ecule pairs **Va/Vb**: −*x* + 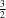, *y* + 1, *z* − 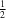 (left) and −*x* + 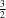, *y* + 1, *z* + 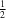 (right). Values of energies by pair: *E*_tot_ = −14.5 kJ mol^−1^, *E*_coul_ = −5.0 kJ mol^−1^, *E*_pol_ = −2.5 kJ mol^−1^, *E*_disp_ = − 23.4 kJ mol^−1^, *E*_rep_ = 16.4 kJ mol^−1^. Inter­action energies were calculated using *PIXEL3.1* (Gavezzotti, 2003 ▸, 2008 ▸) based on densities computed with G09 using the mp2/6–31** level of theory.

**Figure 9:**
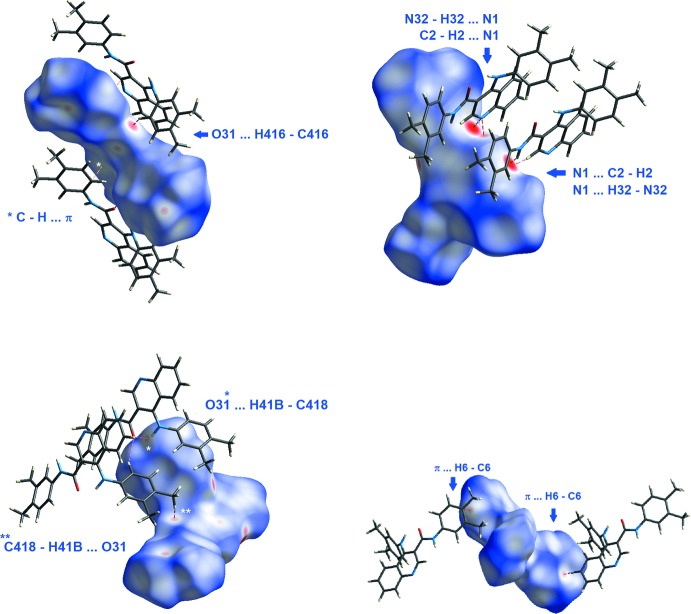
Several faces of the HS plotted over *d*_norm_ for **1** showing the red spots that indicate close contacts between atoms, which are identified in the figures.

Apart from the sub-structural motifs described, there are two extra mol­ecule pairs, identified as **Va**/**Vb** and **VIa**/**VIb**, which are also illustrated in Figs. 7 ▸ and 8 ▸: the two mol­ecules involved are at *x*, *y*, *z* (green-coloured mol­ecule) and −*x* + 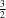, −*y* + 1, *z* − 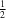/−*x* + 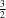, −*y* + 1, *z* + 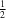 (black-coloured mol­ecule) for **Va/Vb** and *x* − 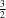, −*y* + 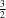, −*z* + 1/*x* − 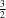, −*y* + 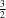, −*z* + 1 for **VIa**/**VIb**. Although these mol­ecules do not exhibit atom⋯atom close contacts, each pair provides a significant contribution to the overall lattice stabilization energy of −14.5 and −11.3 kJ mol^−1^, respectively for **V** and **VI**. The grey mol­ecules drawn in this figure indicate a possible pathway for electronic delocalization within the network of mol­ecules.

## Database survey   

A search of the Cambridge Structural Database (CSD, Version 5.40, November 2019 update; Groom *et al.*, 2016 ▸) for 3,4-disubstituted quinoline with an N—H unit attached to C4 revealed two compounds: SEZJIR (3-acetyl-4-amino­quinoline; Lokaj *et al.*, 2007 ▸) with a carbonyl group attached to C3 and an amino group attached to C4 and PABPUD {4-[3-(*N*,*N*-di­methyl­amino)­propyl­amino]-3-nitro­quinoline; Boyd *et al.*, 1992 ▸} with an amino group attached to C4 and a nitro group attached to C3. In both of these compounds, there is no puckering of the pyridine ring and the quinoline ring system is essentially planar. In both cases, a hydrogen atom forms an intra­molecular hydrogen bond between an amino hydrogen and the carbonyl oxygen in both independent mol­ecules of the asymmetric unit (SEZJIR) or between the amino hydrogen and a nitro group oxygen atom (PABPUD). In both structures, the C(pyridine)⋯N(amino) distances are significantly shorter than those in **1**, *viz*. 1.325 and 1.335 Å for the two mol­ecules in the asymmetric unit of SEZJIR and 1.320 Å in PABPUD. The corresponding value in **1** is 1.364 (3) Å.

A survey of quinoline compounds, with an *R* factor of 10% or less with a C_quinoline_–NH–C_ar­yl/*sp*^3^_ unit attached to C4 of the quinoline moiety gave 56 hits for 63 individual mol­ecules, including **1**. The C_quinoline_—N distances lie in the range 1.319 to 1.438 Å with an average value of 1.360 Å.

The situation is more complex for the N—C_ar­yl/*sp*^3^_ bond and for the C_quinoline_—N—C_ar­yl/*sp*^3^_ angle. A scatterplot of these revealed two populations, one in which the N atom is attached to a benzene ring and the other in which the connection is to an *sp* ^3^ carbon. UNIKUZ [6-(*t*-butyl­sulfon­yl)-*N*-(5-fluoro-1*H*-indazol-3-yl)quinolin-4-amine methanol solvate; Haile *et al.*, 2016 ▸) is included in the first group. The C_ar­yl_—N distances lie in the range 1.396 to 1.438 Å with an average value of 1.418 Å and an average C_quinoline_—N—C_ar­yl/*sp*^3^_ angle of 126.105°. In the second case, the C_ar­yl/*sp*^3^_—N distances lie in the range 1.439 to 1.478 Å with an average value of 1.458 Å, with an average C_quinoline_—N—C_ar­yl/*sp*^3^_ angle of 123.98°.

As noted above, the conformation around the amino N atom is slightly pyramidal. In their paper on bond lengths in organic compounds, Allen *et al.* (2006 ▸) discuss the planarity and pyramidality of amino compounds. They state that for planar N atoms, the mean valence angle is greater than 117.6° while for pyramidal N atoms the mean valence angle lies in the range 108 to 114°. The value for **1** is 117.56°. There are three other structures in this survey which have average valence angles close to but less than 117°. The valence angles are 116.57° in DAMIOT {2,3-bis­[(2,6-di­methyl­phen­yl)sulfan­yl]-*N*-phenyl­quinolin-4-amine; Florke & Egold, 2016 ▸}, 117.41° in MEQKEY (2,4-dianilino-3-ethyl­quinoline; Katritzky *et al.*, 2000 ▸) and 117.04° in OTAMOM {2-(4-meth­oxy­phen­yl)-*N*-[2-(2-phenyl­vin­yl)phen­yl]quinolin-4-amine; Mphahlele & Mphahlele, 2011 ▸}. These four compounds are thus neither strictly planar nor pyramidal.

There are two compounds in the database which have an amide group attached to C3, GICGIL [2-chloro-*N*-(4-fluoro­phen­yl)-6-methyl­quinoline-3-carboxamide; Govender *et al.*, 2018 ▸] and SUZHEB (*N*-isopropyl-6-methyl-2-phenyl­quino­line-3-carboxamide; Benzerka *et al.*, 2010 ▸). In both these compounds, the amide group is inclined to the quinoline moiety, unlike in mol­ecule **1**.

## Synthesis and crystallization   

The title quinolone derivative **1** was synthesized by a one-pot reaction between 4-oxo-1,4-di­hydro­quinoline-3-carb­oxy­lic acid and 3,4-di­methyl­aniline in the presence of POCl_3_ following a procedure described previously (Cagide *et al.*, 2015 ▸). The title compound was obtained in 70% yield and characterized by NMR. It was re-crystallized from di­chloro­methane to yield crystals suitable for X-ray diffraction, m.p. 489–493 K.

NMR data were acquired on a Bruker AMX 400 spectrometer, recorded at room temperature in 5 mm outer-diameter tubes. The samples were prepared in deuterated di­methyl­sulfoxide (DMSO) with tetra­methyl­silane (TMS) as inter­nal reference. Chemical shifts are expressed as δ (ppm) values relative to TMS; coupling constants (*J*) are given in Hz. Atoms are labelled with their numerical designation as per Fig. 1 ▸. See *Supporting Information* for spectra.

**4-(3,4-Di­methyl­anilino)-** ***N*** **-(3,4-di­methyl­phen­yl)quinoline-3-carboxamide**

^1^H NMR (400 MHz, DMSO): 10.16 (1H, *s*, CONH), 9.43 (1H, *s*, NH), 8.82 (1H, *s*, H-2), 8.14 (1H, *dd*, *J* = 1.0, 8.5 Hz, H-8), 7.95 (1H, *dd*, *J* = 0.84, 8.4 Hz, H-5), 7.73 (1H, *ddd*, *J* = 1.0, 6.9, 8.4 Hz, H-6), 7.46 (1H, *ddd*, *J* = 1.0, 6.9, 8.5 Hz, H-7), 7.18 (1H, *d*, *J* = 2.0 Hz, H-412), 7.12 (1H, *dd*, *J* = 2.1, 8.0 Hz H-326), 7.00 (1H, *d*, *J* = 8.0 Hz, H-325), 6.93 (1H, *d*, *J* = 8.0 Hz, H-415), 6.84 (1H, *d*, *J* = 2.1 Hz, H-322), 6.72 (1H, *dd*, *J* = 2.0, 8.0 Hz, H-416), 2.01 (3H, *s*, CH_3_), 2.07 (3H, *s*, CH_3_), 2.16 (6H, *s*, 2 × CH_3_).

^13^C NMR (100 MHz, DMSO): 165.4 (CONH), 149.9 (C-2), 149.1 (C-8*A*), 146.6 (C-4), 140.3 (C-411), 136.5 (C-414), 136.3 (C-321), 135.6 (C-324), 131.2 (C-413), 130.5 (C-323), 130.2 (C-6), 129.6 (C-415), 129.2 (C-5), 129.0 (C-325), 125.0 (C-7), 124.2 (C-8), 121.6 (C-412), 121.5 (C-322), 120.8 (C-4A), 117.8 (C-325), 117.7 (C-416), 114.4 (C-3), 19.5 (CH_3_), 19.3 (CH_3_), 18.7 (CH_3_), 18.5 (CH_3_).

## Refinement   

Crystal data, data collection and structure refinement details are summarized in Table 3 ▸. The H atoms were included in idealized positions and treated as riding atoms: C—H = 0.95–0.98 Å with *U*_iso_(H) = 1.2*U*_eq_(C) or 1.5*U*_eq_(C) for methyl H atoms. Those attached to N and C2 [C—H = 0.96 (3) Å] were refined. The latter was refined since it is involved in a short contact with H32, which is attached to N32. Although in the riding model for H2 the H-atom position is within the highest contour on the difference map, it is not at the centre. In the refined model it is. The H⋯H distances are 1.87 and 1.93 Å for the riding and refined models, respectively. The angles around C2 are N1—C2—C3 = 125.9 (3) and 125.9°(3); N—C2—H2 = 117 and 111.9 (17)° and C3—C2—H2 = 117 and 122.2 (17)° for riding and refined H atoms, respectively. In the case of H32, the N32—H32 distance changes from 0.89 (3) to 0.90 (4) Å and the angle C31—N32—H32 changes from 120 (2) to 119 (2)° for riding to refined, respectively, which are really insignificant shifts. Hence, in this case the short contact does induce a shift in the angular position of H2 from its calculated position.

## Supplementary Material

Crystal structure: contains datablock(s) I, global. DOI: 10.1107/S2056989020000298/zl2767sup1.cif

Structure factors: contains datablock(s) I. DOI: 10.1107/S2056989020000298/zl2767Isup2.hkl

Spectra. DOI: 10.1107/S2056989020000298/zl2767sup3.tif

CCDC reference: 1879928

Additional supporting information:  crystallographic information; 3D view; checkCIF report

## Figures and Tables

**Figure 8:**
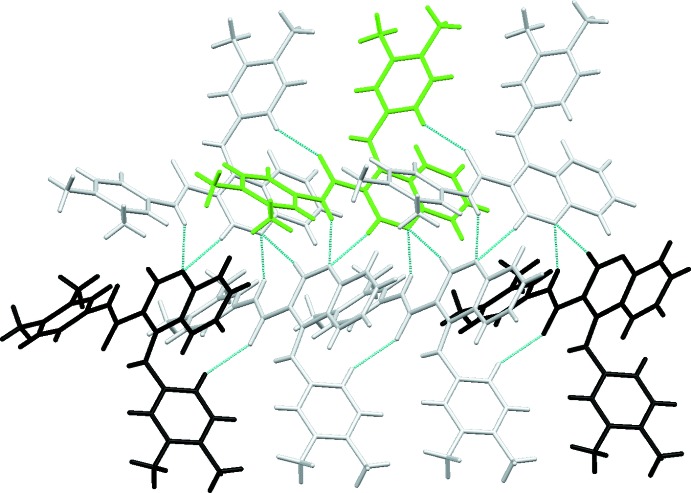
Mol­ecule pairs **VIa/VIb**, (*x* − 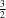, −*y* + 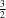, −*z* + 1 (left) and *x* − 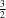, −*y* + 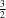, −*z* + 1(right). Values of energies by pair: *E*_tot_ = −11.3 kJ mol^−1^, *E*_coul_ = −3.3 kJ mol^−1^, *E*_pol_ = −2.2 kJ mol^−1^, *E*_disp_ = −15.5 kJ mol^−1^, *E*_rep_ = 9.6 kJ mol^−1^. Inter­action energies were calculated using *PIXEL3.1* (Gavezzotti, 2003 ▸, 2008 ▸) based on densities computed with G09 using the mp2/6–31** level of theory.

**Table 1:** Hydrogen-bond geometry (Å, °) *Cg* is the centroid of the N1/C2–C4/C4*A*/C8*A* ring.

*D*—H⋯*A*	*D*—H	H⋯*A*	*D*⋯*A*	*D*—H⋯*A*
N41—H41⋯O31	0.84 (4)	1.93 (3)	2.635 (3)	142 (3)
C326—H326⋯O31	0.95	2.40	2.887 (3)	112
N32—H32⋯N1^i^	0.90 (4)	2.07 (4)	2.891 (3)	150 (3)
C2—H2⋯N1^i^	0.96 (3)	2.55 (3)	3.477 (4)	163 (2)
C416—H416⋯O31^ii^	0.95	2.39	3.252 (4)	150
C326—H326⋯*Cg* ^iii^	0.95	2.82	3.398 (3)	120

Symmetry codes: (i) 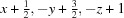; (ii) 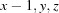; (iii) 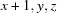.

**Table 2:** Percentages for atom⋯atom close contacts

Compound	H⋯H	H⋯O/O⋯H	H⋯C/C⋯H	C⋯C	O⋯C/C⋯O	N⋯N	H⋯N/N⋯H	C⋯N/N⋯C
**1**	58.4	4.3	27.0	2.5	0.6	0.2	6.5	0.5

**Table 3:** Experimental details

Crystal data	
Chemical formula	C_26_H_25_N_3_O
*M*_r_	395.49
Crystal system, space group	Orthorhombic, *P*2_1_2_1_2_1_
Temperature (K)	100
*a*, *b*, *c* (Å)	6.2502 (3), 15.7915 (6), 20.7395 (9)
*V* (Å^3^)	2046.99 (15)
*Z*	4
Radiation type	Mo *K*α
μ (mm^−1^)	0.08
Crystal size (mm)	0.30 × 0.05 × 0.01

Data collection	
Diffractometer	Rigaku FRE+ equipped with VHF Varimax confocal mirrors and an AFC12 goniometer and HyPix 6000 detector
Absorption correction	Multi-scan (*CrysAlis PRO*; Rigaku OD, 2018 ▸)
*T*_min_, *T*_max_	0.487, 1.000
No. of measured, independent and observed [*I* > 2σ(*I*)] reflections	28997, 3754, 3390
*R*_int_	0.089
(sin θ/λ)_max_ (Å^−1^)	0.602

Refinement	
*R*[*F* ^2^ > 2σ(*F* ^2^)], *wR*(*F* ^2^), *S*	0.044, 0.094, 1.08
No. of reflections	3754
No. of parameters	287
H-atom treatment	H atoms treated by a mixture of independent and constrained refinement
Δρ_max_, Δρ_min_ (e Å^−3^)	0.22, −0.20
Absolute structure	Flack *x* determined using 1238 quotients [(*I* ^+^)−(*I* ^−^)]/[(*I* ^+^)+(*I* ^−^)] (Parsons *et al.*, 2013 ▸)
Absolute structure parameter	0.2 (10)

Computer programs: *CrysAlis PRO* (Rigaku OD, 2018 ▸), *SHELXT* (Sheldrick, 2015*a* ▸), *ShelXle* (Hübschle *et al.*, 2011 ▸), *SHELXL2014/7* (Sheldrick, 2015*b* ▸), *OSCAIL* (McArdle *et al.*, 2004 ▸), *Mercury* (Macrae *et al.*, 2006 ▸) and *PLATON* (Spek, 2020 ▸).

## supplementary crystallographic information

### Crystal data

**Table:**

C_26_H_25_N_3_O	*D*_x_ = 1.283 Mg m^−^^3^
*M**_r_* = 395.49	Mo *K*α radiation, λ = 0.71075 Å
Orthorhombic, *P*2_1_2_1_2_1_	Cell parameters from 5133 reflections
*a* = 6.2502 (3) Å	θ = 1.6–27.0°
*b* = 15.7915 (6) Å	µ = 0.08 mm^−^^1^
*c* = 20.7395 (9) Å	*T* = 100 K
*V* = 2046.99 (15) Å^3^	Needle, yellow
*Z* = 4	0.30 × 0.05 × 0.01 mm
*F*(000) = 840	

### Data collection

**Table:**

Rigaku FRE+ equipped with VHF Varimax confocal mirrors and an AFC12 goniometer and HyPix 6000 detector diffractometer	3754 independent reflections
Radiation source: Rotating Anode, Rigaku FRE+	3390 reflections with *I* > 2σ(*I*)
Confocal mirrors, VHF Varimax monochromator	*R*_int_ = 0.089
Detector resolution: 10 pixels mm^-1^	θ_max_ = 25.4°, θ_min_ = 1.6°
profile data from ω–scans	*h* = −7→7
Absorption correction: multi-scan (CrysAlis PRO; Rigaku OD, 2018)	*k* = −19→19
*T*_min_ = 0.487, *T*_max_ = 1.000	*l* = −24→24
28997 measured reflections	

### Refinement

**Table:**

Refinement on *F*^2^	Secondary atom site location: structure-invariant direct methods
Least-squares matrix: full	Hydrogen site location: mixed
*R*[*F*^2^ > 2σ(*F*^2^)] = 0.044	H atoms treated by a mixture of independent and constrained refinement
*wR*(*F*^2^) = 0.094	*w* = 1/[σ^2^(*F*_o_^2^) + (0.0402*P*)^2^ + 0.5091*P*] where *P* = (*F*_o_^2^ + 2*F*_c_^2^)/3
*S* = 1.08	(Δ/σ)_max_ < 0.001
3754 reflections	Δρ_max_ = 0.22 e Å^−^^3^
287 parameters	Δρ_min_ = −0.20 e Å^−^^3^
0 restraints	Absolute structure: Flack *x* determined using 1238 quotients [(*I*^+^)-(*I*^-^)]/[(*I*^+^)+(*I*^-^)] (Parsons *et al.*, 2013)
Primary atom site location: structure-invariant direct methods	Absolute structure parameter: 0.2 (10)

### Special details

**Table:**

Geometry. All esds (except the esd in the dihedral angle between two l.s. planes) are estimated using the full covariance matrix. The cell esds are taken into account individually in the estimation of esds in distances, angles and torsion angles; correlations between esds in cell parameters are only used when they are defined by crystal symmetry. An approximate (isotropic) treatment of cell esds is used for estimating esds involving l.s. planes.

### Fractional atomic coordinates and isotropic or equivalent isotropic displacement parameters (Å^2^)

**Table:**

	*x*	*y*	*z*	*U*_iso_*/*U*_eq_	
O31	0.5597 (3)	0.49964 (12)	0.49759 (10)	0.0221 (5)	
N1	−0.0092 (4)	0.69133 (14)	0.44267 (11)	0.0174 (5)	
N32	0.5344 (4)	0.62696 (15)	0.54659 (11)	0.0162 (5)	
H32	0.470 (5)	0.678 (2)	0.5477 (15)	0.028 (9)*	
N41	0.2497 (4)	0.44714 (15)	0.42129 (13)	0.0195 (5)	
H41	0.368 (6)	0.441 (2)	0.4400 (16)	0.026 (9)*	
C2	0.1722 (5)	0.66921 (17)	0.47005 (13)	0.0161 (6)	
H2	0.233 (5)	0.7147 (17)	0.4948 (14)	0.014 (7)*	
C3	0.2727 (4)	0.58912 (17)	0.46478 (13)	0.0154 (6)	
C4	0.1727 (5)	0.52774 (16)	0.42608 (13)	0.0158 (6)	
C5	−0.1008 (5)	0.50493 (18)	0.33862 (13)	0.0196 (6)	
H5	−0.037379	0.452194	0.327658	0.024*	
C4A	−0.0119 (4)	0.55400 (17)	0.38887 (13)	0.0161 (6)	
C6	−0.2769 (5)	0.53228 (19)	0.30555 (15)	0.0237 (7)	
H6	−0.332691	0.499128	0.271220	0.028*	
C7	−0.3757 (5)	0.60896 (19)	0.32201 (15)	0.0241 (7)	
H7	−0.504081	0.625506	0.300995	0.029*	
C8	−0.2880 (5)	0.65997 (19)	0.36813 (14)	0.0209 (6)	
H8	−0.352964	0.712802	0.377996	0.025*	
C8A	−0.1007 (5)	0.63448 (17)	0.40134 (13)	0.0160 (6)	
C31	0.4680 (4)	0.56876 (16)	0.50328 (13)	0.0158 (6)	
C321	0.7023 (4)	0.61744 (16)	0.59179 (13)	0.0146 (6)	
C322	0.6840 (5)	0.66241 (17)	0.64919 (14)	0.0173 (6)	
H322	0.559581	0.695626	0.656408	0.021*	
C323	0.8416 (4)	0.66032 (18)	0.69622 (14)	0.0184 (6)	
C324	1.0277 (5)	0.61192 (18)	0.68531 (13)	0.0181 (6)	
C325	1.0432 (5)	0.56727 (18)	0.62795 (14)	0.0191 (6)	
H325	1.167921	0.534397	0.620254	0.023*	
C326	0.8845 (4)	0.56865 (17)	0.58145 (14)	0.0168 (6)	
H326	0.899709	0.536669	0.542929	0.020*	
C327	0.8115 (5)	0.70814 (19)	0.75834 (15)	0.0250 (7)	
H32A	0.934273	0.745612	0.765419	0.037*	
H32B	0.800307	0.667948	0.794181	0.037*	
H32C	0.680368	0.741992	0.755852	0.037*	
C328	1.2038 (5)	0.6077 (2)	0.73465 (15)	0.0254 (7)	
H32D	1.253251	0.665128	0.744633	0.038*	
H32E	1.323221	0.574413	0.717458	0.038*	
H32F	1.149786	0.580841	0.774012	0.038*	
C411	0.1222 (5)	0.37148 (17)	0.41833 (14)	0.0194 (6)	
C412	0.2064 (5)	0.30036 (17)	0.38929 (13)	0.0202 (7)	
H412	0.346385	0.302560	0.371508	0.024*	
C413	0.0886 (5)	0.22452 (18)	0.38552 (13)	0.0192 (6)	
C414	−0.1149 (5)	0.22182 (18)	0.41275 (14)	0.0208 (7)	
C415	−0.1955 (5)	0.29255 (18)	0.44308 (14)	0.0228 (7)	
H415	−0.333552	0.290071	0.462158	0.027*	
C416	−0.0793 (5)	0.36751 (18)	0.44637 (14)	0.0200 (6)	
H416	−0.137324	0.415606	0.467629	0.024*	
C417	0.1826 (5)	0.14893 (18)	0.35187 (16)	0.0275 (7)	
H41D	0.090414	0.132683	0.315709	0.041*	
H41E	0.193326	0.101625	0.382290	0.041*	
H41F	0.325447	0.163163	0.335672	0.041*	
C418	−0.2461 (5)	0.14148 (19)	0.40803 (16)	0.0281 (7)	
H41A	−0.384750	0.150125	0.429087	0.042*	
H41B	−0.169739	0.095098	0.429390	0.042*	
H41C	−0.268722	0.127195	0.362554	0.042*	

### Atomic displacement parameters (Å^2^)

**Table:**

	*U*^11^	*U*^22^	*U*^33^	*U*^12^	*U*^13^	*U*^23^
O31	0.0180 (10)	0.0128 (9)	0.0356 (12)	0.0031 (8)	−0.0017 (9)	−0.0058 (9)
N1	0.0155 (12)	0.0137 (11)	0.0229 (13)	−0.0001 (10)	0.0022 (11)	−0.0008 (10)
N32	0.0136 (12)	0.0114 (11)	0.0236 (13)	0.0014 (10)	−0.0003 (10)	−0.0012 (10)
N41	0.0152 (13)	0.0120 (12)	0.0314 (14)	0.0002 (10)	−0.0025 (12)	−0.0058 (10)
C2	0.0173 (14)	0.0107 (13)	0.0202 (15)	−0.0031 (12)	0.0028 (12)	−0.0018 (11)
C3	0.0133 (14)	0.0137 (13)	0.0194 (14)	−0.0012 (12)	0.0052 (12)	−0.0011 (11)
C4	0.0152 (14)	0.0139 (13)	0.0184 (15)	−0.0010 (11)	0.0042 (12)	−0.0010 (11)
C5	0.0203 (15)	0.0159 (14)	0.0226 (15)	−0.0033 (13)	0.0013 (13)	−0.0032 (12)
C4A	0.0146 (14)	0.0146 (14)	0.0193 (14)	−0.0032 (12)	0.0040 (12)	0.0013 (11)
C6	0.0273 (17)	0.0212 (15)	0.0224 (15)	−0.0067 (14)	−0.0068 (14)	−0.0013 (12)
C7	0.0203 (16)	0.0232 (16)	0.0287 (17)	−0.0014 (13)	−0.0065 (13)	0.0028 (13)
C8	0.0195 (15)	0.0171 (14)	0.0260 (16)	0.0008 (13)	0.0010 (13)	0.0024 (12)
C8A	0.0164 (14)	0.0138 (13)	0.0179 (14)	−0.0031 (12)	0.0048 (12)	−0.0003 (11)
C31	0.0145 (14)	0.0123 (12)	0.0208 (15)	−0.0020 (12)	0.0057 (12)	0.0000 (12)
C321	0.0136 (14)	0.0103 (13)	0.0200 (14)	−0.0020 (11)	0.0009 (12)	0.0035 (11)
C322	0.0163 (14)	0.0118 (13)	0.0238 (16)	−0.0002 (12)	0.0036 (12)	0.0004 (11)
C323	0.0171 (14)	0.0180 (14)	0.0202 (15)	−0.0030 (13)	0.0036 (12)	0.0041 (11)
C324	0.0159 (15)	0.0152 (14)	0.0232 (15)	−0.0035 (12)	0.0031 (12)	0.0057 (12)
C325	0.0138 (14)	0.0168 (14)	0.0267 (16)	0.0018 (12)	0.0048 (12)	0.0032 (12)
C326	0.0151 (14)	0.0148 (13)	0.0205 (15)	−0.0018 (11)	0.0034 (12)	−0.0011 (12)
C327	0.0246 (16)	0.0234 (16)	0.0269 (17)	0.0001 (14)	0.0022 (14)	−0.0033 (13)
C328	0.0186 (16)	0.0316 (17)	0.0261 (16)	−0.0015 (14)	0.0003 (13)	0.0025 (13)
C411	0.0241 (16)	0.0132 (13)	0.0208 (15)	−0.0013 (12)	−0.0066 (13)	−0.0002 (12)
C412	0.0225 (16)	0.0174 (14)	0.0209 (16)	0.0005 (13)	−0.0013 (13)	0.0001 (12)
C413	0.0279 (17)	0.0128 (14)	0.0171 (15)	0.0022 (12)	−0.0057 (13)	−0.0007 (11)
C414	0.0223 (16)	0.0205 (15)	0.0197 (16)	−0.0021 (12)	−0.0065 (13)	0.0028 (12)
C415	0.0198 (15)	0.0252 (16)	0.0235 (16)	−0.0019 (13)	−0.0022 (13)	0.0035 (12)
C416	0.0206 (15)	0.0150 (14)	0.0245 (16)	0.0005 (12)	−0.0025 (13)	−0.0018 (12)
C417	0.0307 (17)	0.0171 (15)	0.0347 (18)	−0.0001 (14)	0.0016 (15)	−0.0046 (13)
C418	0.0298 (18)	0.0213 (16)	0.0333 (18)	−0.0062 (14)	−0.0042 (15)	0.0036 (13)

### Geometric parameters (Å, º)

**Table:**

O31—C31	1.238 (3)	C323—C327	1.505 (4)
N1—C2	1.315 (4)	C324—C325	1.386 (4)
N1—C8A	1.367 (4)	C324—C328	1.505 (4)
N32—C31	1.350 (3)	C325—C326	1.384 (4)
N32—C321	1.416 (4)	C325—H325	0.9500
N32—H32	0.90 (4)	C326—H326	0.9500
N41—C4	1.364 (3)	C327—H32A	0.9800
N41—C411	1.437 (4)	C327—H32B	0.9800
N41—H41	0.84 (4)	C327—H32C	0.9800
C2—C3	1.416 (4)	C328—H32D	0.9800
C2—H2	0.96 (3)	C328—H32E	0.9800
C3—C4	1.405 (4)	C328—H32F	0.9800
C3—C31	1.494 (4)	C411—C412	1.379 (4)
C4—C4A	1.449 (4)	C411—C416	1.389 (4)
C5—C6	1.367 (4)	C412—C413	1.408 (4)
C5—C4A	1.413 (4)	C412—H412	0.9500
C5—H5	0.9500	C413—C414	1.392 (4)
C4A—C8A	1.411 (4)	C413—C417	1.503 (4)
C6—C7	1.401 (4)	C414—C415	1.377 (4)
C6—H6	0.9500	C414—C418	1.514 (4)
C7—C8	1.365 (4)	C415—C416	1.390 (4)
C7—H7	0.9500	C415—H415	0.9500
C8—C8A	1.417 (4)	C416—H416	0.9500
C8—H8	0.9500	C417—H41D	0.9800
C321—C322	1.391 (4)	C417—H41E	0.9800
C321—C326	1.391 (4)	C417—H41F	0.9800
C322—C323	1.387 (4)	C418—H41A	0.9800
C322—H322	0.9500	C418—H41B	0.9800
C323—C324	1.410 (4)	C418—H41C	0.9800
			
C2—N1—C8A	117.2 (2)	C326—C325—C324	122.7 (3)
C31—N32—C321	126.6 (2)	C326—C325—H325	118.7
C31—N32—H32	119 (2)	C324—C325—H325	118.7
C321—N32—H32	114 (2)	C325—C326—C321	119.2 (3)
C4—N41—C411	125.7 (2)	C325—C326—H326	120.4
C4—N41—H41	112 (2)	C321—C326—H326	120.4
C411—N41—H41	115 (2)	C323—C327—H32A	109.5
N1—C2—C3	125.9 (3)	C323—C327—H32B	109.5
N1—C2—H2	111.9 (17)	H32A—C327—H32B	109.5
C3—C2—H2	122.2 (17)	C323—C327—H32C	109.5
C4—C3—C2	117.6 (3)	H32A—C327—H32C	109.5
C4—C3—C31	121.3 (2)	H32B—C327—H32C	109.5
C2—C3—C31	120.9 (2)	C324—C328—H32D	109.5
N41—C4—C3	121.9 (3)	C324—C328—H32E	109.5
N41—C4—C4A	120.6 (2)	H32D—C328—H32E	109.5
C3—C4—C4A	117.4 (2)	C324—C328—H32F	109.5
C6—C5—C4A	120.9 (3)	H32D—C328—H32F	109.5
C6—C5—H5	119.5	H32E—C328—H32F	109.5
C4A—C5—H5	119.5	C412—C411—C416	119.5 (3)
C8A—C4A—C5	118.3 (3)	C412—C411—N41	119.0 (3)
C8A—C4A—C4	118.3 (2)	C416—C411—N41	121.5 (2)
C5—C4A—C4	123.3 (3)	C411—C412—C413	121.2 (3)
C5—C6—C7	120.4 (3)	C411—C412—H412	119.4
C5—C6—H6	119.8	C413—C412—H412	119.4
C7—C6—H6	119.8	C414—C413—C412	118.8 (3)
C8—C7—C6	120.3 (3)	C414—C413—C417	121.4 (3)
C8—C7—H7	119.9	C412—C413—C417	119.8 (3)
C6—C7—H7	119.9	C415—C414—C413	119.6 (3)
C7—C8—C8A	120.3 (3)	C415—C414—C418	120.7 (3)
C7—C8—H8	119.9	C413—C414—C418	119.6 (3)
C8A—C8—H8	119.9	C414—C415—C416	121.5 (3)
N1—C8A—C4A	122.8 (3)	C414—C415—H415	119.3
N1—C8A—C8	117.7 (3)	C416—C415—H415	119.3
C4A—C8A—C8	119.5 (3)	C411—C416—C415	119.4 (3)
O31—C31—N32	121.4 (3)	C411—C416—H416	120.3
O31—C31—C3	121.1 (2)	C415—C416—H416	120.3
N32—C31—C3	117.4 (2)	C413—C417—H41D	109.5
C322—C321—C326	118.8 (3)	C413—C417—H41E	109.5
C322—C321—N32	116.8 (2)	H41D—C417—H41E	109.5
C326—C321—N32	124.3 (2)	C413—C417—H41F	109.5
C323—C322—C321	122.1 (3)	H41D—C417—H41F	109.5
C323—C322—H322	119.0	H41E—C417—H41F	109.5
C321—C322—H322	119.0	C414—C418—H41A	109.5
C322—C323—C324	119.1 (3)	C414—C418—H41B	109.5
C322—C323—C327	120.1 (3)	H41A—C418—H41B	109.5
C324—C323—C327	120.8 (3)	C414—C418—H41C	109.5
C325—C324—C323	118.1 (3)	H41A—C418—H41C	109.5
C325—C324—C328	120.7 (3)	H41B—C418—H41C	109.5
C323—C324—C328	121.2 (3)		
			
C8A—N1—C2—C3	−5.5 (4)	C2—C3—C31—N32	5.0 (4)
N1—C2—C3—C4	1.1 (4)	C31—N32—C321—C322	−150.3 (3)
N1—C2—C3—C31	−174.0 (3)	C31—N32—C321—C326	31.7 (4)
C411—N41—C4—C3	141.0 (3)	C326—C321—C322—C323	0.2 (4)
C411—N41—C4—C4A	−41.6 (4)	N32—C321—C322—C323	−177.9 (2)
C2—C3—C4—N41	−175.7 (3)	C321—C322—C323—C324	0.8 (4)
C31—C3—C4—N41	−0.6 (4)	C321—C322—C323—C327	−178.3 (3)
C2—C3—C4—C4A	6.8 (4)	C322—C323—C324—C325	−0.9 (4)
C31—C3—C4—C4A	−178.1 (2)	C327—C323—C324—C325	178.1 (3)
C6—C5—C4A—C8A	−4.1 (4)	C322—C323—C324—C328	179.8 (3)
C6—C5—C4A—C4	179.2 (3)	C327—C323—C324—C328	−1.2 (4)
N41—C4—C4A—C8A	172.4 (3)	C323—C324—C325—C326	0.1 (4)
C3—C4—C4A—C8A	−10.1 (4)	C328—C324—C325—C326	179.4 (3)
N41—C4—C4A—C5	−10.8 (4)	C324—C325—C326—C321	0.8 (4)
C3—C4—C4A—C5	166.7 (3)	C322—C321—C326—C325	−1.0 (4)
C4A—C5—C6—C7	−1.5 (4)	N32—C321—C326—C325	176.9 (2)
C5—C6—C7—C8	4.7 (5)	C4—N41—C411—C412	155.0 (3)
C6—C7—C8—C8A	−2.2 (4)	C4—N41—C411—C416	−27.8 (4)
C2—N1—C8A—C4A	1.7 (4)	C416—C411—C412—C413	2.3 (4)
C2—N1—C8A—C8	−175.7 (2)	N41—C411—C412—C413	179.6 (3)
C5—C4A—C8A—N1	−170.9 (3)	C411—C412—C413—C414	−1.1 (4)
C4—C4A—C8A—N1	6.0 (4)	C411—C412—C413—C417	178.3 (3)
C5—C4A—C8A—C8	6.4 (4)	C412—C413—C414—C415	−0.5 (4)
C4—C4A—C8A—C8	−176.7 (2)	C417—C413—C414—C415	−179.9 (3)
C7—C8—C8A—N1	174.1 (3)	C412—C413—C414—C418	178.7 (3)
C7—C8—C8A—C4A	−3.4 (4)	C417—C413—C414—C418	−0.6 (4)
C321—N32—C31—O31	−3.1 (4)	C413—C414—C415—C416	1.0 (4)
C321—N32—C31—C3	173.8 (2)	C418—C414—C415—C416	−178.3 (3)
C4—C3—C31—O31	6.9 (4)	C412—C411—C416—C415	−1.8 (4)
C2—C3—C31—O31	−178.1 (3)	N41—C411—C416—C415	−179.1 (3)
C4—C3—C31—N32	−170.0 (3)	C414—C415—C416—C411	0.2 (4)

### Hydrogen-bond geometry (Å, º)

*Cg* is the centroid of the N1/C2–C4/C4*A*/C8*A* ring.

**Table:**

*D*—H···*A*	*D*—H	H···*A*	*D*···*A*	*D*—H···*A*
N41—H41···O31	0.84 (4)	1.93 (3)	2.635 (3)	142 (3)
C326—H326···O31	0.95	2.40	2.887 (3)	112
N32—H32···N1^i^	0.90 (4)	2.07 (4)	2.891 (3)	150 (3)
C2—H2···N1^i^	0.96 (3)	2.55 (3)	3.477 (4)	163 (2)
C416—H416···O31^ii^	0.95	2.39	3.252 (4)	150
C326—H326···*Cg*^iii^	0.95	2.82	3.398 (3)	120

Symmetry codes: (i) *x*+1/2, −*y*+3/2, −*z*+1; (ii) *x*−1, *y*, *z*; (iii) *x*+1, *y*, *z*.
