# Supplementary figures and images for: The synthesis, crystal structure and Hirshfeld analysis of 4-(3,4-di­methyl­anilino)-N-(3,4-di­methyl­phen­yl)quinoline-3-carboxamide
Source: Acta Crystallogr E Crystallogr Commun. 2020 Jan 17;76(Pt 2):201–7. doi: 10.1107/S2056989020000298 (PMC7001829; doi:10.1107/S2056989020000298)

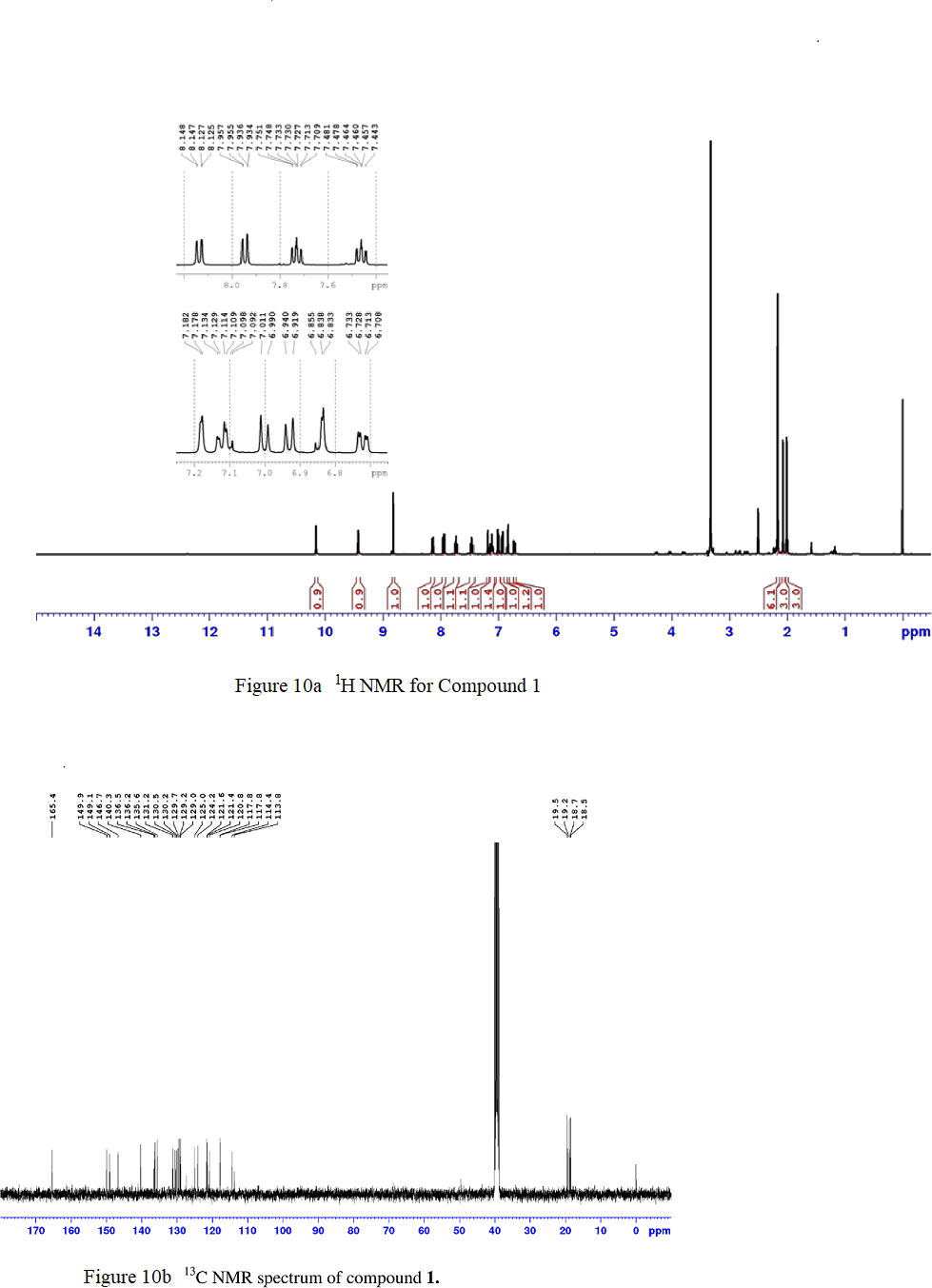

Supplement: Supplementary file 3 [file e-76-00201-sup3.tif]
